# Prospective Association Between Brazilian Primary Oral Health Care Models and Preventive Dental Visits and Risk Behaviours: A 5‐Year Cohort Study Among Young Adults

**DOI:** 10.1111/cdoe.70075

**Published:** 2026-05-22

**Authors:** Jocelaine Vieira Vione, Letícia Donato Comim, Nathália Costa de Castro, Julio Eduardo do Amaral Zenkner, Orlando Luiz do Amaral Júnior, Luana Severo Alves

**Affiliations:** ^1^ Department of Restorative Dentistry, School of Dentistry Federal University of Santa Maria Santa Maria Brazil; ^2^ Department of Stomatology, School of Dentistry Federal University of Santa Maria Santa Maria Rio Grande do Sul Brazil

**Keywords:** dental health services, health inequities, longitudinal studies, oral health, social determinants of health

## Abstract

**Objective:**

To evaluate the prospective association between the absence and different models of primary oral health care in Brazil (traditional units and Family Health Strategy [FHS] teams) and preventive dental visits and risk behaviours (smoking initiation and increase in alcohol consumption) among young individuals.

**Methods:**

This 5‐year prospective cohort study derived from a population‐based cross‐sectional survey conducted in 2018 that evaluated the oral health conditions of 1197 15‐ to 19‐year‐old adolescents from southern Brazil. In 2022–2024, follow‐up assessments were conducted (mean time interval 5 ± 0.5 years). Socio‐demographic characteristics, the use of dental services (including the reason for the last dental visit), behavioural habits (smoking and alcohol consumption) and participants' address were collected at baseline and follow‐up. The outcomes were last dental visit for prevention, smoking initiation and increase in alcohol consumption. The main predictor variable was public dental services coverage in the neighbourhood (absent, primary health care or FHS including a dental team) obtained from official data. Unadjusted and adjusted multilevel Poisson regression models were performed to estimate relative risk (RR) and 95% confidence intervals (CI).

**Results:**

Five hundred and seventy individuals were re‐evaluated (47.6% retention). After adjusting for individual‐level variables, participants residing in neighbourhoods with FHS including a dental team were more likely to visit a dentist for preventive purposes (RR: 1.59; 95% CI: 1.29–1.97), and had a lower risk for smoking initiation (RR: 0.45; 95% CI: 0.21–0.97), and an increase in alcohol consumption (RR: 0.80; 95% CI: 0.67–0.97) compared to areas without coverage. Coverage by traditional primary health care showed no significant association with the study outcomes.

**Conclusion:**

Brazil's FHS model with integrated oral health teams outperforms traditional dental services in promoting preventive care and reducing risk behaviours among young adults.

## 1 Introduction

Brazil's Unified Health System (SUS) was established to provide universal and equitable access to health services, guided by the principles of universality, comprehensiveness, regionalization and social participation. Its structure integrates primary, secondary and tertiary levels of care, aiming to reduce historical disparities in service provision and health outcomes [1]. To strengthen primary health care, the Family Health Program was launched in 1994, later expanded into the Family Health Strategy (FHS) [2]. Oral health was incorporated into primary care in 2000, and in 2004 the National Oral Health Policy was launched, proposing a care model centred on the individual rather than on disease [3]. This integration represented a shift from a predominantly surgical restorative approach to one that emphasizes prevention, continuity of care and actions targeted at community needs [4].

Two main public dental service models currently operate within SUS: traditional primary care units and FHS units with oral health teams [5]. Traditional units are organized as clinic‐based services focused primarily on individual treatment demands and less connected to community settings. In contrast, FHS oral health teams are embedded within a broader territorial framework, in which interdisciplinary groups of dentists, nurses, physicians and community health workers share responsibility for defined geographic areas [5, 6]. Their actions extend beyond clinical interventions to include household visits, school‐based programmes and community prevention initiatives. These territorialized strategies aim to address social determinants of health, acknowledging that cultural norms, family structures and neighbourhood conditions influence both service use and risk behaviours [7]. Longitudinality and continuity of care are therefore core principles of this model, distinguishing it from episodic, demand‐driven care.

Determining whether such organizational and cultural distinctions in service delivery are associated with measurable behavioural outcomes has important implications for shaping oral health and primary care policies worldwide [8]. If FHS coverage increases preventive dental attendance and reduces adoption of harmful behaviours, these findings could inform expansion and optimization of community‐oriented, territorially structured care models, supporting efficient allocation of resources and enhancing population health [9]. Smoking and alcohol consumption are established common risk factors for major oral health conditions, including periodontal disease and oral cancer, reinforcing the relevance of examining these behaviours within primary care contexts [10]. Understanding how different organizational approaches influence health behaviours provides evidence to guide policy adaptation in other countries seeking to integrate oral health into primary care frameworks [9, 11].

This prospective cohort study aimed to evaluate the prospective association between the absence and different models of primary oral health care in Brazil (traditional units and FHS teams) and preventive dental visits and risk behaviours (smoking initiation and increase in alcohol consumption) among young individuals. It was hypothesized that coverage by FHS teams would be associated with greater use of preventive services and lower incidence of risk behaviours, given its comprehensive and community‐oriented approach.

## 2 Methods

### 2.1 Ethical Aspects

The study was approved by the Research Ethics Committee of the Federal University of Santa Maria (CAAE 43938021.0.0000.5346) and is in accordance with The Code of Ethics of the World Medical Association (Declaration of Helsinki) for experiments involving humans. All participants signed a written informed consent form, received a report of their oral health status and were referred to dental treatment when needed.

### 2.2 Study Design and Sample

This is a prospective cohort study of adolescents conducted in Santa Maria, a mid‐sized city located in southern Brazil. This municipality has a territorial area of 1780.194 km^2^, with an estimated population of 271 735 inhabitants in 2022 [12]. The urban area is divided into 42 neighbourhoods distributed across eight administrative regions. In 2018, the estimated population coverage by oral health services in primary health care was 22.4% [13].

In 2018, a cross‐sectional study was carried out to assess the oral health conditions of adolescents aged from 15 to 19, who were attending private and public high schools. For the sample selection of the study, adolescents born in the years 1999–2003, enrolled in the regular school year and attending any school period (morning, afternoon or night) were considered eligible. The number of participants selected from each school was proportional to the number of enrolled students. Individuals using fixed orthodontic appliances or those presenting with special needs were not considered eligible. A list of all eligible students was compiled for each school, and a random sample was selected using a table of random numbers (www.random.org). The minimum sample size required was calculated based on the following parameters: prevalence of 50% (‘worst case scenario’), 95% confidence interval (CI), power of 80% and precision level of 3%. It was estimated that 1066 students would be required, to which a non‐participation rate of 50% was added, totalling 1600 adolescents to be invited to participate. All 37 urban schools were invited, of which 31 agreed to participate (22 public and 9 private). A total of 1197 adolescents aged 15–19 years were included in the cross‐sectional study.

At the 5‐year follow‐up, all individuals assessed at baseline were considered eligible to participate. Different search strategies were implemented to achieve the largest possible number of participants for this follow‐up. Initially, contact was attempted through phone numbers, email and social media platforms such as Facebook, Instagram and WhatsApp. If these attempts were unsuccessful, home visits were conducted at the addresses provided in the questionnaires. Once contact was established, individuals were invited to participate in this second phase of the study. When they agreed, data collection was conducted either at the clinics of the School of Dentistry of the Federal University of Santa Maria or at the participant's residence, depending on their availability and preference.

### 2.3 Data Collection

This study includes data collected at two time points: baseline, conducted from March to November 2018, and follow‐up, carried out from December 2022 to April 2024 (mean ± standard deviation of 5 ± 0.5 years). At both time points, participants self‐completed a structured questionnaire, which addressed demographic and socio‐economic characteristics, the use of dental services and behavioural habits. The questionnaire was tested and adjusted to allow better comprehension. Two members of the research team were present in the field to organize logistics and distribute the forms, but they did not provide any additional information or guidance during completion.

Demographic variables included sex (male or female), age (15, 16, 17 or 18–19) and skin colour, which was collected based on the Brazilian criteria [14] and subsequently dichotomized as white or non‐white. Socio‐economic status (SES) was collected using the Brazilian Economic Classification Criterion [15] and households were then classified into low (≤ 16 points, corresponding to social class DE), mid‐low (≥ 17 to ≤ 22 points, corresponding to social class C2), mid‐high (≥ 23 to ≤ 28 points, corresponding to social class C1) or high (≥ 29 points, corresponding to social classes A, B1 and B2) SES. School type (public or private) and residential mobility from baseline to follow‐up (change of neighbourhood: no or yes) were also collected.

Regarding the use of dental services, participants were asked about the reason for the last dental visit (check‐up/prevention, tooth ache, extraction, fillings or others), smoking (no or yes), and the frequency of consumption of alcoholic beverages (never, rarely, sometimes, often or every day). This questionnaire also collected information about the participant's residential address, allowing for the identification of the neighbourhood based on official sources [16]. Additionally, information on the public dental services available in each neighbourhood of the municipality was collected at baseline using official data, and public dental service coverage was classified as absent, primary health care or FHS including an oral health team.

### 2.4 Statistical Analysis

Data were analysed using the STATA software (Stata 14.2; Stata Corporation, College Station, USA). A weight variable based on the probability of selection and population distribution according to sex and SES was used to adjust for the potential bias in the population estimates [17]. The chi‐squared test was performed to compare characteristics between the followed participants and those lost to follow‐up.

The outcomes of this study were last visit to the dentist for prevention, smoking initiation and increase in alcohol consumption, all of them defined as binary variables. To define the first outcome, the question ‘what was the reason for your last visit to the dentist?’ was used, and participants who answered ‘check‐up/prevention’ in both observation periods (baseline and follow‐up) were classified as ‘yes’. This definition was adopted to capture maintenance of preventive care behaviour over time, rather than isolated utilization at follow‐up. Since longitudinality and continuity of care are central attributes of the FHS, sustained engagement in preventive visits was considered a proxy of stable preventive behaviour within the primary care context. This operationalization does not assess behavioural change per se, but persistence of preventive service use across the follow‐up period. Therefore, those who chose this option only at baseline, only at follow‐up or in none of the observation periods were classified as ‘no’. Smoking initiation was defined as ‘yes’ for participants who were non‐smokers at baseline but smokers at follow‐up. Increase in alcohol consumption was defined as ‘yes’ when the frequency reported at follow‐up was higher than the frequency reported at baseline.

The main predictor variable was public dental service coverage, as previously described. All predictor variables were measured at baseline.

Public dental service coverage was defined according to the availability of services in the participant's neighbourhood of residence at baseline, serving as a contextual proxy for local availability of primary oral health care. Although the FHS is formally organized at the municipal level and operates through defined catchment areas that do not necessarily correspond to administrative neighbourhood boundaries, neighbourhood‐level classification was adopted to approximate the immediate residential context.

**FIGURE 1:**
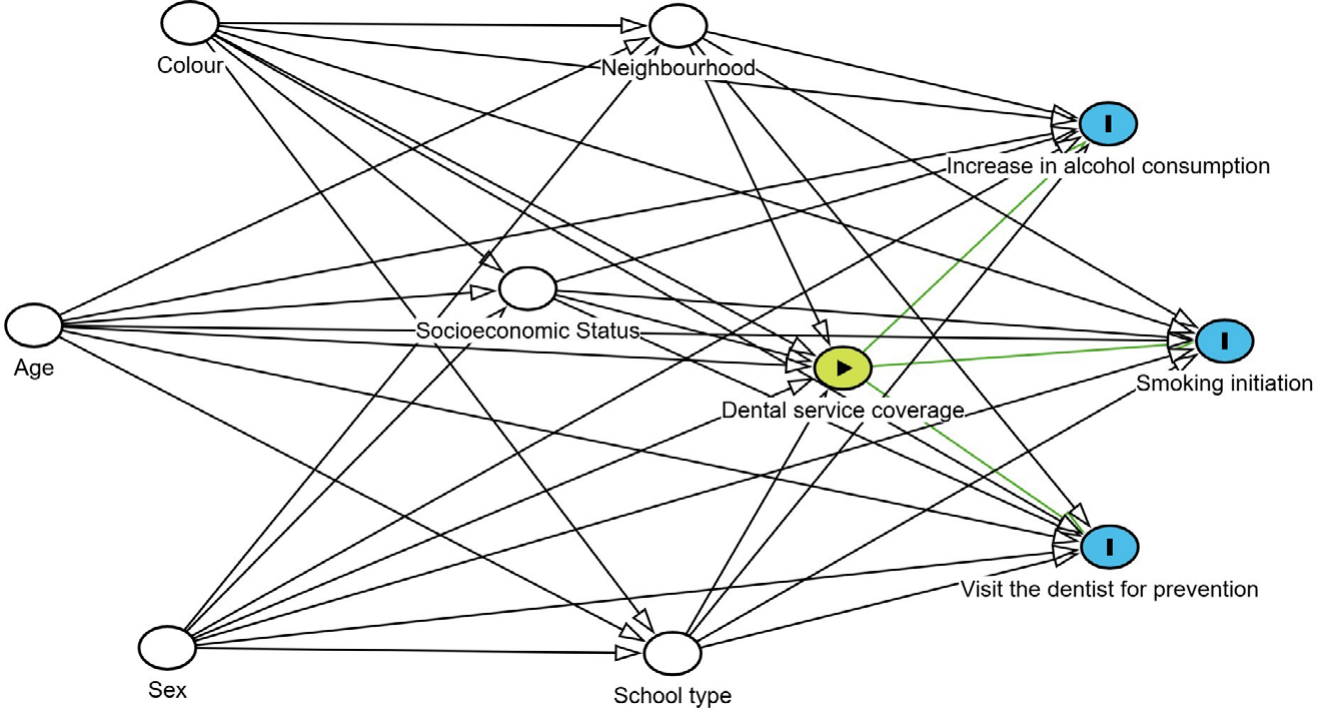
Directed acyclic graph illustrating the hypothesized causal structure between public dental services coverage and preventive dental visits, smoking initiation and increase in alcohol consumption among young adults.

Multilevel Poisson regression models were specified with individuals at level 1 and administrative regions of the city at level 2, including a random intercept for region. Administrative regions were used as the contextual unit due to the limited number of participants per neighbourhood, which could compromise model stability and variance estimation at the neighbourhood level. These regions represent broader territorial units within the municipality that share urban infrastructure and local health service organization. This modelling strategy allows estimation of contextual variance at a higher territorial level while examining associations between neighbourhood‐level service availability and individual outcomes. Unadjusted and adjusted relative risks (RR) and 95% confidence intervals (CI) were estimated. To guide confounder selection, we constructed a directed acyclic graph (DAG) a priori using DAGitty (Version 3.1, available at http://www.dagitty.net/). The DAG explicitly modelled the assumed causal relationships between the contextual exposure (public dental services coverage), the outcomes and all measured covariates, allowing identification of the minimally sufficient adjustment set to block all backdoor paths (Figure 1).

## 3 Results

**FIGURE 2:**
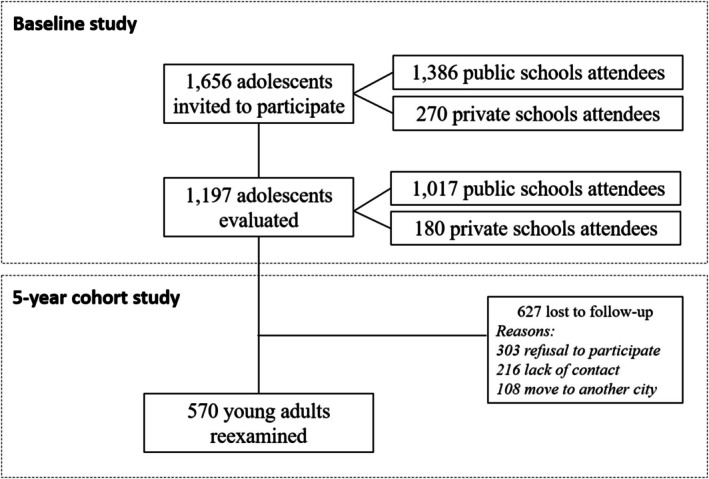
Study flow chart.

Of the 1197 adolescents assessed at baseline, 570 were reassessed at the 5‐year follow‐up, resulting in a cohort retention rate of 47.6%. The primary reasons for loss to follow‐up were refusal to continue participating in the study (*n* = 303), loss of contact with participants (*n* = 216) and relocation to another city (*n* = 108) (Figure 2). When comparing baseline characteristics of those who remained in the study and those lost to follow‐up, statistically significant differences were observed regarding age, skin colour and SES. The proportion of younger individuals, those with white skin and those from higher SES was significantly greater among participants who remained in the study compared to those lost to follow‐up. No significant differences were observed regarding sex.

As six participants had an incomplete address that prevented the definition of their neighbourhood of residence and two others lived in nearby cities, the final sample included 562 young adults. The sample distribution and the proportion of participants who visited the dentist for preventive purposes, initiated smoking and increased alcohol consumption according to contextual and individual variables are presented in Table 1. Among the 562 participants, 24.9% reported visiting the dentist for preventive purposes (*n* = 149). Smoking initiation occurred in 11.5% of the sample (*n* = 57), while an increase in alcohol consumption was reported by 47.8% of the participants (*n* = 272). Regarding the coverage of public dental service in the neighbourhood, the incidence of smoking initiation was lower among residents of areas covered by the FHS with an oral health team (5%) relative to the other two categories (absent, 13%; primary health care, 16.5%). Similarly, the increase in alcohol consumption was lower in areas covered by the FHS with an oral health team (41.5%) than in neighbourhoods without public dental services (51.8%). No significant differences were detected regarding the use of dental serviced for preventive purposes in this preliminary analysis.

**TABLE 1:** Sample distribution, and the proportion (95% confidence interval) of participants who visited the dentist for preventive purposes, started smoking and increased alcohol consumption by predictor variables.

	*n* (%)	Visit the dentist for prevention	Smoking initiation	Increase in alcohol consumption
Contextual variable				
Public dental services				
Absent	223 (39.7)	23.2 (17.5–28.9)^a^	13.0 (8.1–17.9)^a^	51.8 (44.9–58.7)^a^
Primary health care	153 (27.2)	23.2 (16.4–30.0)^a^	16.5 (10.1–22.9)^a^	49.2 (40.9–57.5)^ab^
FHS + dental team	186 (33.1)	28.7 (22.0–35.4)^a^	5.0 (1.5–8.5)^b^	41.5 (34.2–48.8)^b^
Individual variables				
Sex				
Male	234 (41.6)	26.5 (20.6–32.3)^a^	16.6 (11.2–21.9)^a^	47.2 (40.3–54.0)^a^
Female	328 (58.4)	23.7 (19.1–28.3)^a^	7.3 (4.4–10.2)^b^	48.3 (42.9–53.8)^a^
Age				
15	144 (25.6)	27.5 (20.0–35.0)^ab^	9.9 (4.8–15.1)^a^	56.3 (47.9–64.7)^a^
16	183 (32.6)	31.7 (24.7–38.7)^b^	13.4 (7.8–18.9)^a^	46.4 (38.8–53.9)^a^
17	171 (30.4)	18.2 (12.5–24.0)^a^	9.7 (4.7–14.8)^a^	49.4 (41.6–57.3)^a^
18–19	64 (11.4)	18.5 (8.6–28.4)^a^	14.0 (4.6–23.4)^a^	29.5 (17.9–41.1)^b^
Skin colour^a^				
Non‐white	149 (27.1)	23.2 (16.2–30.2)^a^	12.2 (6.6–17.8)^a^	42.9 (34.7–51.2)^a^
White	400 (72.9)	26.3 (21.9–30.7)^a^	10.7 (7.3–14.1)^a^	50.1 (44.9–55.2)^a^
Socio‐economic status				
Low	79 (14.1)	16.7 (8.3–25.1)^a^	19.4 (10.3–28.5)^a^	41.9 (30.9–52.9)^a^
Mid‐low	130 (23.1)	21.2 (14.0–28.4)^a^	9.5 (4.1–15.0)^ab^	49.8 (40.9–58.7)^a^
Mid‐high	139 (24.7)	26.5 (19.1–33.9)^ab^	14.3 (8.5–20.1)^a^	49.9 (41.5–58.2)^a^
High	214 (38.1)	32.9 (26.6–39.2)^b^	5.6 (2.5–8.7)^b^	48.3 (41.6–55.0)^a^
School type				
Public	445 (79.2)	23.6 (19.6–27.7)^a^	11.5 (8.2–14.8)^a^	45.1 (40.3–50.0)^a^
Private	117 (20.8)	30.8 (22.3–39.3)^a^	11.2 (4.9–17.5)^a^	60.0 (50.8–69.2)^b^
Neighbourhood change				
No	391 (9.6)	25.8 (21.3–30.2)^a^	11.4 (7.9–14.9)^a^	48.8 (43.6–54.0)^a^
Yes	171 (30.4)	23.1 (16.8–29.5)^a^	11.6 (6.2–17.0)^a^	45.7 (37.9–53.5)^a^
Total	562 (100)	24.9 (21.3–28.6)	11.5 (8.5–14.4)	47.8 (43.5–52.1)

*Note:* Different letters indicate statistically significant difference between categories (*p* < 0.05, adjusted Wald test).

Abbreviation: FHS, Family Health Strategy.

^a^ Missing data.

The unadjusted Poisson regression models are presented in Table 2. Dental service coverage by the FHS including an oral health team was associated with a higher frequency of preventive dental visits (RR: 1.69; 95% CI: 1.37–2.08; *p* ≤ 0.001) and a lower risk for smoking initiation (RR: 0.41; 95% CI: 0.18–0.92; *p* = 0.03) when compared to neighbourhoods without public dental services. No association was identified between FHS coverage and an increase in alcohol consumption in the unadjusted analysis (RR: 0.82; 95% CI: 0.58–1.18; *p* = 0.29).

**TABLE 2:** Unadjusted multilevel Poisson regression models investigating the prospective association between public dental services coverage and the study outcomes (visit the dentist for preventive purposes, smoking initiation and increase in alcohol consumption).

	Visit the dentist for prevention		Smoking initiation		Increase in alcohol consumption	
	RR (95% CI)	*p*	RR (95% CI)	*p*	RR (95% CI)	*p*
Contextual variable						
Public dental services						
Absent	1.00		1.00		1.00	
Primary health care	1.05 (0.75–1.48)	0.763	1.37 (0.63–2.96)	0.424	0.95 (0.80–1.13)	0.581
Family health strategy	1.69 (1.37–2.08)	≤ 0.001	0.41 (0.18–0.92)	0.031	0.82 (0.58–1.18)	0.287
Individual variables						
Sex						
Male	1.00		1.00		1.00	
Female	0.94 (0.67–1.32)	0.722	0.44 (0.35–0.55)	≤ 0.001	1.04 (0.93–1.16)	0.459
Age						
15	1.00		1.00		1.00	
16	1.23 (0.86–1.74)	0.254	1.42 (0.57–3.50)	0.451	0.85 (0.66–1.09)	0.210
17	0.69 (0.43–1.13)	0.140	1.12 (0.56–2.21)	0.751	0.89 (0.77–1.03)	0.118
18–19	0.74 (0.38–1.43)	0.370	1.58 (0.97–2.59)	0.065	0.54 (0.43–0.68)	≤ 0.001
Skin colour						
Non‐white	1.00		1.00		1.00	
White	1.13 (0.95–1.34)	0.174	0.93 (0.64–1.35)	0.705	1.14 (0.94–1.39)	0.172
Socio‐economic status						
Low	1.00		1.00		1.00	
Mid‐low	1.30 (0.79–2.16)	0.300	0.44 (0.15–1.30)	0.138	1.14 (0.86–1.51)	0.369
Mid‐high	1.71 (1.30–2.24)	≤ 0.001	0.68 (0.43–1.09)	0.108	1.17 (0.87–1.56)	0.292
High	2.14 (1.26–3.65)	0.005	0.28 (0.11–0.69)	0.005	1.11 (0.83–1.49)	0.466
School type						
Public	1.00		1.00		1.00	
Private	1.30 (0.94–1.80)	0.107	0.86 (0.32–2.32)	0.771	1.34 (1.18–1.52)	< 0.001
Neighbourhood change						
No	1.00		1.00		1.00	
Yes	0.90 (0.77–1.06)	0.203	0.82 (0.49–1.38)	0.458	0.91 (0.65–1.29)	0.616

Abbreviations: CI, confidence interval; RR, relative risk.

**FIGURE 3:**
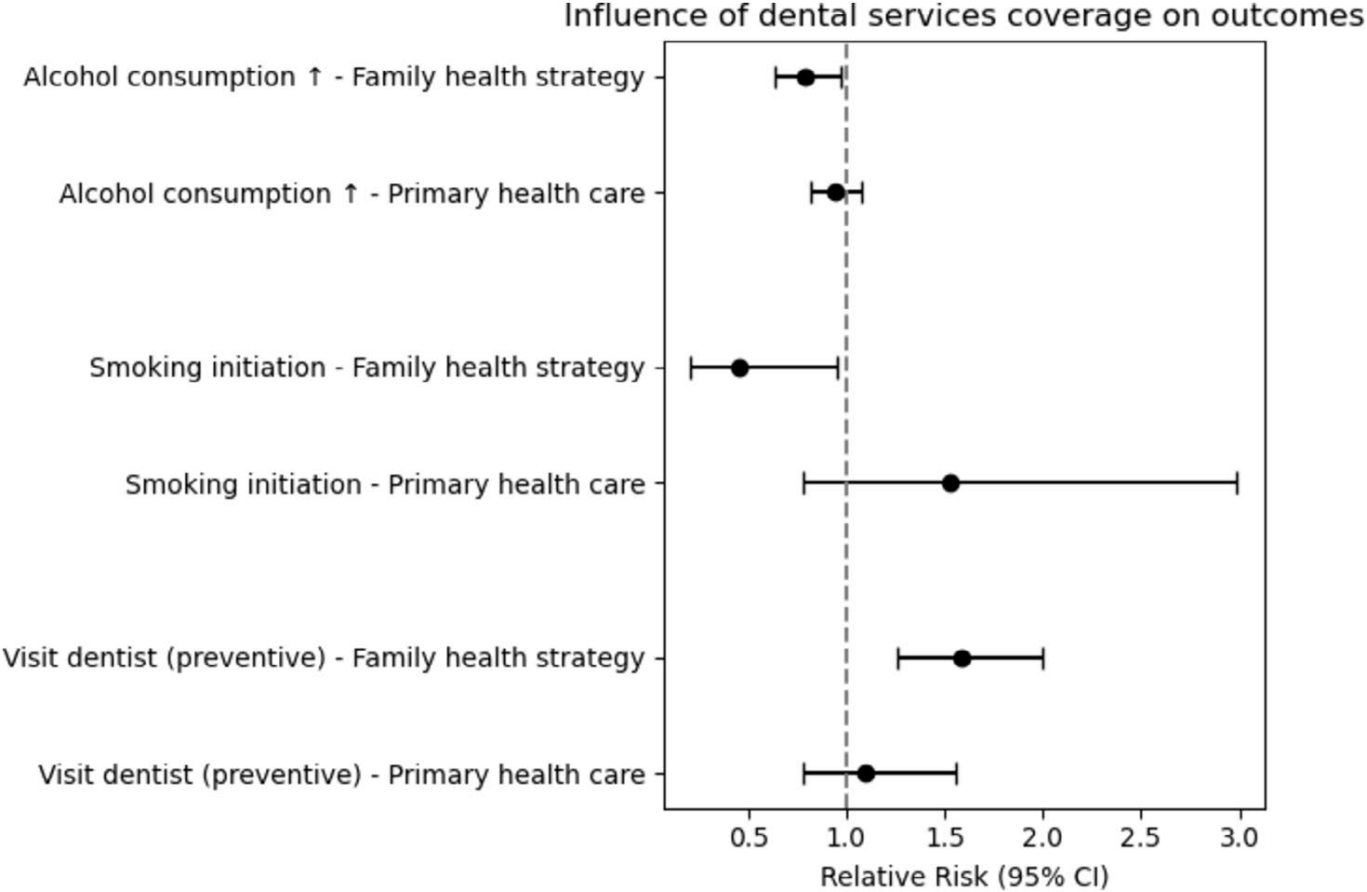
Visual representation of adjusted relative risks (RR) and 95% confidence intervals for the prospective association between public dental services coverage and the study outcomes (visit the dentist for preventive purposes, smoking initiation and increase in alcohol consumption) among young adults. Results correspond to those shown in Table 3. The dashed line indicates RR = 1.

After adjusting the estimates for sex, age, skin colour, SES, type of school and neighbourhood change (Table 3), participants who lived in neighbourhoods covered by the FHS with an oral health team had a 59% greater risk for visiting a dentist for preventive reasons than their counterparts who lived in neighbourhoods without public dental services (RR: 1.59; 95% CI: 1.29–1.97; *p* < 0.001). Additionally, FHS coverage afforded protection against smoking initiation and increase in alcohol consumption. Young adults living in neighbourhoods covered by the FHS with an oral health team had a 55% and 20% lower risk of starting smoking (RR: 0.45; 95% CI: 0.21–0.97; *p* = 0.043) and increasing frequency of consumption of alcoholic beverages (RR: 0.80; 95% CI: 0.67–0.97; *p* = 0.022), respectively, than those living in neighbourhoods without dentists in the public service. On the other hand, coverage through traditional primary care was not significantly associated with any of the outcomes evaluated, in either the unadjusted or the adjusted models. These associations are illustrated in Figure 3, which presents the adjusted RR estimates and 95% CIs for public dental service coverage (traditional primary health care and FHS with oral health teams) in relation to preventive dental visits, smoking initiation and increased alcohol consumption among young adults. This figure provides a visual summary of the multilevel Poisson regression results presented in the text. The vertical dashed line indicates the null effect (RR = 1).

**TABLE 3:** Adjusted multilevel Poisson regression models investigating the prospective association between dental services coverage and the study outcomes (visit the dentist for preventive purposes, smoking initiation and increase in alcohol consumption).

	Visit the dentist for prevention		Smoking initiation		Increase in alcohol consumption	
	RR (95% CI)	*p*	RR (95% CI)	*p*	RR (95% CI)	*p*
Contextual variable						
Dental services coverage						
Absent	1.00		1.00		1.00	
Primary health care	1.10 (0.78–1.54)	0.575	1.54 (0.78–3.06)	0.216	0.94 (0.83–1.07)	0.362
Family health strategy	1.59 (1.29–1.97)	< 0.001	0.45 (0.21–0.97)	0.043	0.80 (0.67–0.97)	0.022

*Note:* Estimates are adjusted for sex, age, skin colour, socio‐economic status, type of school and neighbourhood change.

Abbreviations: CI, confidence interval; RR, relative risk.

## 4 Discussion

This longitudinal study provides evidence that Brazil's FHS with integrated oral health teams significantly improves preventive dental utilization and reduces risk behaviours among young adults compared to traditional primary care models or areas without public dental services. Our findings demonstrate that the FHS's community‐based approach can lead to better oral health outcomes while simultaneously addressing broader health behaviours during a critical developmental period.

Beyond the Brazilian context, these findings contribute to the literature on primary care organization and oral health inequalities. Previous research has demonstrated that access to preventive dental services and adoption of health‐promoting behaviours are socially patterned and influenced by structural characteristics of health systems [18, 19]. From this perspective, territorially organized primary care models may reduce barriers to preventive utilization by increasing service availability and by strengthening continuity, trust and community linkage [20]. The absence of significant associations for traditional primary care in this study reinforces the notion that organizational attributes of care delivery, rather than service presence alone, are central to influencing behavioural trajectories and to reducing social gradients in oral health [21].

The superior performance of FHS in promoting preventive dental visits highlights the effectiveness of its territorial, interdisciplinary model. Unlike traditional clinic‐based services that focus on individual treatment needs, FHS teams implement comprehensive strategies including home visits, school programmes and community education [22]. Importantly, the FHS model emphasizes longitudinality and continuity of care, which may explain why adolescents in FHS‐covered areas were more likely to sustain preventive dental visits over the 5‐year follow‐up, rather than seeking care only sporadically. This aligns with Brazil's National Oral Health Policy objectives to shift from a curative to preventive‐promotional paradigm [22]. Our results are consistent with a previous study showing that FHS coverage improves child oral health outcomes, and extend this evidence to young adult populations where longitudinal data are limited [23].

The protective effect of FHS coverage against smoking initiation and alcohol consumption escalation suggests its interventions address common risk factors for both oral and general health [24]. This likely occurs through multiple pathways: health education during dental consultations, early identification of risk behaviours by community health workers and the programme's emphasis on social determinants of health [10, 24]. These findings support the common risk factor approach, demonstrating how integrated primary care models can simultaneously improve multiple health outcomes. The lack of significant associations with traditional dental services underscores that mere clinical availability, without FHS's proactive community engagement, may be insufficient to modify health behaviours [25].

From a policy perspective, these results strongly justify continued investment in expanding FHS oral health teams. The programme's dual impact on oral health service utilization and risk behaviours makes it particularly cost‐effective for adolescent and young adult populations [26]. Future implementation should focus on strengthening intersectoral collaborations between oral health teams, schools and social services to maximize preventive impacts. Policymakers should also consider targeted training for FHS teams in adolescent‐specific health promotion strategies [27].

Study limitations include potential selection bias due to loss to follow‐up, which may have influenced the representativeness of the final sample despite the use of statistical weighting to adjust for observed demographic differences. Baseline differences between retained and non‐retained participants in age, skin colour and socio‐economic status suggest differential attrition, which may have introduced selection bias if these characteristics were associated with exposure or outcomes, and this type of bias is not corrected by weighting procedures based on the initial sampling design. Residual confounding by unmeasured individual or contextual variables, including neighbourhood‐level factors, cannot be fully excluded despite multilevel adjustment. Measurement error in self‐reported behaviours such as smoking initiation and alcohol consumption may have introduced misclassification bias. In addition, public dental service coverage was measured only at baseline and may not fully reflect potential changes in territorial coverage or residential mobility during follow‐up, limiting causal interpretation of the observed associations. Finally, the generalizability of the findings may be limited to similar urban Brazilian settings with comparable health service organization.

In addition, public dental service coverage was measured ecologically at the neighbourhood level and attributed to all residents based on baseline address. This approach assumes relative homogeneity in service availability and organization within neighbourhoods, an assumption that may not hold, given documented variability in the functioning of oral health teams under the FHS. Moreover, exposure was assessed only at baseline and does not account for changes in service coverage over time or for participants who moved during follow‐up. These limitations likely resulted in non‐differential misclassification of exposure, which tends to bias effect estimates towards the null. Therefore, the associations observed for FHS coverage, particularly the protective effects on smoking initiation and alcohol consumption, may be conservative estimates of the true effects.

This study provides compelling evidence that Brazil's FHS model with integrated oral health teams outperforms traditional dental services in promoting preventive care and reducing risk behaviours among young adults. The findings underscore the importance of community‐oriented, territorially based primary care models for achieving comprehensive health outcomes. As Brazil continues to strengthen its unified health system, these results support prioritizing FHS expansion as an effective strategy for addressing oral health inequalities and their social determinants during critical life stages.

## Author Contributions

Jocelaine Vieira Vione collected the data, drafted and revised the manuscript. Letícia Donato Comim designed the data collection instruments, collected the data, drafted and revised the manuscript. Nathália Costa de Castro collected the data, drafted and revised the manuscript. Julio Eduardo do Amaral Zenkner conceptualized and designed the study and critically revised the manuscript. Orlando Luiz do Amaral Júnior conceptualized the study, wrote the main manuscript text and critically revised the manuscript. Luana Severo Alves conceptualized and designed the study, coordinated and supervised data collection, carried out the analyses, drafted and critically revised the manuscript. All authors approved the final manuscript as submitted and agreed to be accountable for all aspects of the work.

## Funding

We acknowledge the support provided by the Federal University of Santa Maria, the National Coordination of Post‐graduate Education (CAPES), and the National Council for Scientific and Technological Development (CNPq), Ministry of Education, Brazil (funding code 001).

## Ethics Statement

The study protocol was approved by the Research Ethics Committee of the Federal University of Santa Maria (#4.619.015). This research was conducted in full accordance with ethical principles, including the World Medical Association Declaration of Helsinki and the additional requirements from Brazil.

## Consent

All participants signed a written informed consent form. They received a report of their oral health status after clinical examination and were referred to dental treatment when needed.

## Conflicts of Interest

The authors declare no conflicts of interest.

## Data Availability

The data that support the findings of this study are available from the corresponding author upon reasonable request.
